# A Dual-Mode Bandpass Filter with Multiple Controllable Transmission-Zeros Using T-Shaped Stub-Loaded Resonators

**DOI:** 10.1155/2014/572360

**Published:** 2014-02-09

**Authors:** Zh. Yao, C. Wang, N. Y. Kim

**Affiliations:** RFIC Center, Kwangwoon University, 447-1 Wolgye-dong, Nowon-ku, Seoul 139-701, Republic of Korea

## Abstract

A dual-mode broadband bandpass filter (BPF) with multiple controllable transmission-zeros using T-shaped stub-loaded resonators (TSSLRs) is presented. Due to the symmetrical plane, the odd-even-mode theory can be adopted to characterize the BPF. The proposed filter consists of a dual-mode TSSLR and two modified feed-lines, which introduce two capacitive and inductive source-load (S-L) couplings. Five controllable transmission zeros (TZs) can be achieved for the high selectivity and the wide stopband because of the tunable amount of coupling capacitance and inductance. The center frequency of the proposed BPF is 5.8 GHz, with a 3 dB fraction bandwidth of 8.9%. The measured insertion and return losses are 1.75 and 28.18 dB, respectively. A compact size and second harmonic frequency suppression can be obtained by the proposed BPF with S-L couplings.

## 1. Introduction

There has been tremendous growth in unlicensed national information infrastructure (U-NII) radio band applications, including radio frequency identification (RFID) and WiMax applications [1–3]. Planar BPFs, with their compact size, low insertion loss, stringent frequency selectivity, and wide stopband, are in high demand among these applications. Generally, dual-mode resonators can be widely used to meet this demand because of their advantageous compact size. Wolff [4] first demonstrated dual-mode resonators in 1972. Later, dual-mode microstrip resonators became attractive because each can be used as two independent resonant circuits, allowing a compact topology size. Several other types of dual-mode resonators have been proposed, including circular ring [5], open stub [6], circular disk [7], and triangular patch [8] resonators. However, these resonators have only one TZ near the passband.

Selectivity is another critical issue for BPFs and can be improved by introducing TZs near the passband. Usually, nonadjacent coupling, including cross coupling and S-L coupling, can generate TZs by providing a multipath effect [9]. A wide stopband can be obtained by these out-of-band TZs. The cascade trisection and cascade quadruplet are two of the most commonly used cross coupling topologies, but both lead to a large circuit size. S-L couplings have also been widely used in microstrip BPFs. In [10, 11], circular and rectangular dual-mode filters with S-L coupling were proposed. Those filters introduce two TZs at the stopband utilizing the S-L coupling.

In this study, we present the design for a dual-mode BPF for RFID or WiMax applications. The characteristics of the proposed TSSLRs are analyzed by dual-mode theory. Two capacitive and inductive S-L couplings are introduced to generate more TZs without increasing the implementation area. The proposed BPF shows a high out-of-band rejection level and second harmonic suppression.

## 2. C-Band BPF Design

**Figure 1:**
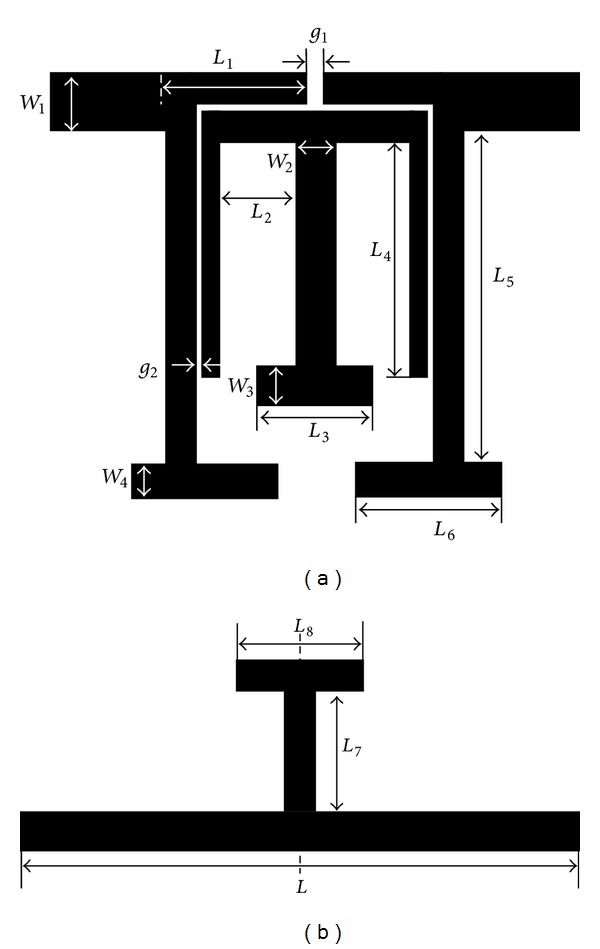
(a) Layout of the dual-mode BPF and (b) schematic view of the proposed TSSLR.

**Figure 2:**
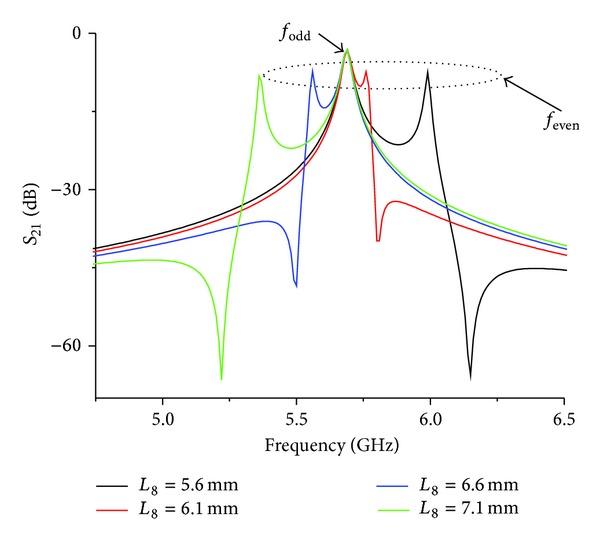
Simulated return loss (*S*_21_) of the TSSLR.

**Figure 3:**
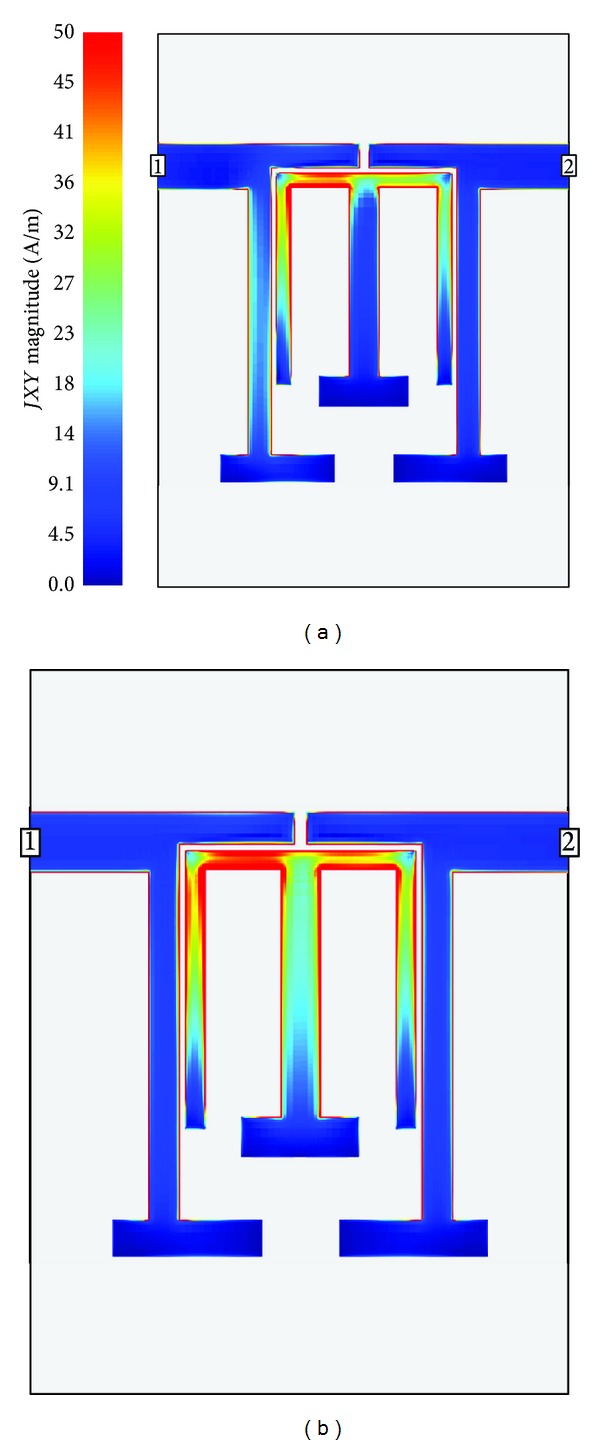
The current distribution in the proposed BPF at resonant frequency: (a) odd-mode and (b) even-mode.

The layout of the microstrip planar BPF is shown in Figure 1(a), which is composed of a dual-mode TSSLR, as shown in Figure 1(b). The characteristics of the TSSLR can be explained using the odd-even-mode theory. The full-wave electromagnetic (EM) Sonnet simulator was used to simulate the response of the dual-mode TSSLR, and the simulation results are illustrated in Figure 2. As seen, the even-mode resonant frequency varies with the parameter *L*_8_. We change  *L*_8_ from 5.6 mm to 7.1 mm, the even-mode is shifted from a lower to a higher frequency, while the odd-mode is not changed. Furthermore, Figures 3(a) and 3(b) show the current distribution of the two modes, respectively. There is a voltage null along the middle of the TSSLR under the odd-mode operation. In this case, the loaded element has no effect on the odd-mode characteristic, which is the reason why the parameter *L*_8_ cannot shift the odd-mode. Under the even-mode operation, a virtual open circuit occurs along the axis of symmetry, so almost all of the current is concentrated on the proposed stub. Hence, the characteristic impedance of the proposed TSSLRs can accurately control both the odd- and even-mode resonant frequencies.

**Figure 4:**
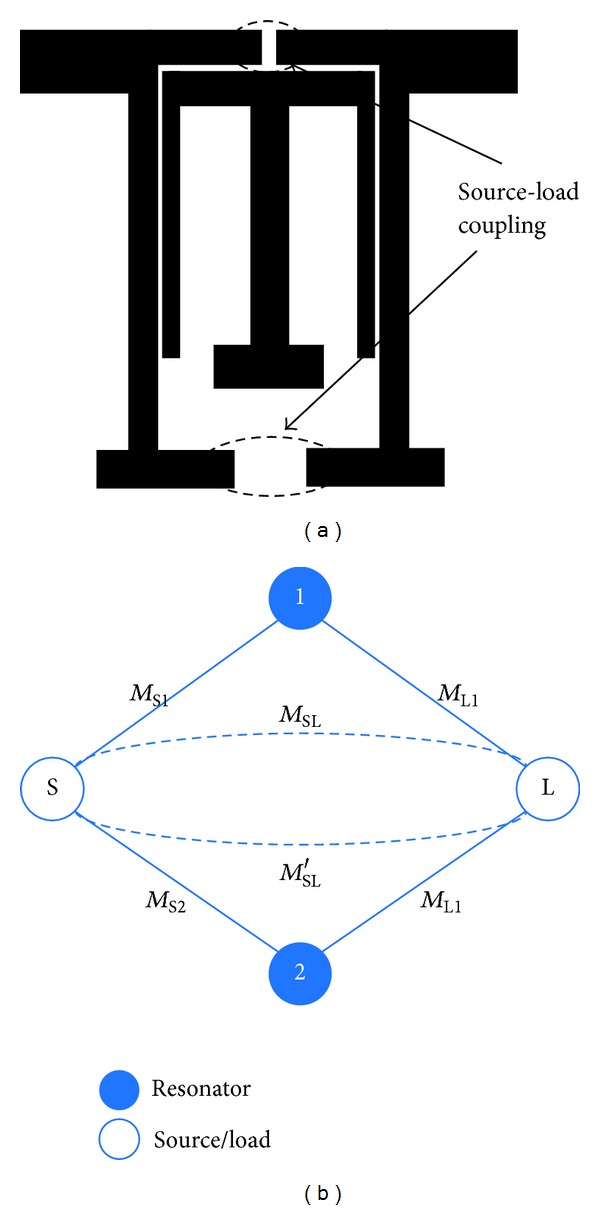
Coupling topology of the proposed BPF.

Figure 4 shows the proposed capacitive and inductive S-L couplings and coupling topology, where the empty disks represent the source and load, respectively. The solid disks represent the odd-mode and even-mode. The input signal is coupled to two modes using coupled-line coupling [12] (represented by the solid lines), as is the output signal. The dashed lines represent the S-L couplings. The coupling topology is analyzed by means of the coupling matrix *M*, given by [13, 14]

(1) $$\begin{array}{c} M=\left[\begin{array}{cc} \begin{array}{cc} 0 & M_{S1}\\ M_{S1} & M_{22} \end{array} & \begin{array}{cc} M_{S2} & M_{SL}\\ 0 & M_{1L} \end{array}\\ \begin{array}{cc} M_{S2} & 0\\ M_{SL} & M_{1L} \end{array} & \begin{array}{cc} M_{22} & M_{2L}\\ M_{2L} & 0 \end{array} \end{array}\right]. \end{array}$$

An explicit mathematical expression with respect to the coupling elements and the TZs is provided by

(2) $$\begin{array}{c} \Omega =\frac{\left(M_{11}M_{S2}^{2}-M_{22}M_{S1}^{2}\right)}{\left(M_{S2}^{2}-M_{S1}^{2}\right)}, \end{array}$$

where  |*M*_*S*1_| > |*M*_*S*2_| is always true for this structure because the coupling strength between the odd-mode and the external feed-line is always larger than that of the even-mode. Therefore, this structure will at least have one TZ at finite frequency. Moreover, the resonant frequencies of the odd-mode and even-mode are related to *M*_*S*1_ and *M*_*S*2_, which can be expressed as

(3) $$\begin{array}{c} f_{\text{odd}}=f_{0}\left(1-\frac{M_{11}\times \Delta f}{2f_{0}}\right), \end{array}$$

(4) $$\begin{array}{c} f_{\text{even}}=f_{0}\left(1-\frac{M_{22}\times \Delta f}{2f_{0}}\right), \end{array}$$

where *f*_0_ and Δ*f* are the center frequency and the bandwidth, respectively. Using (1) and (3), a new expression of *Ω* can be obtained as

(5) $$\begin{array}{c} \Omega \times \left(f_{\text{even}}-f_{0}\right)=\frac{-M_{22}\times \Delta f}{2}\times \frac{\left(M_{11}M_{S2}^{2}-M_{22}M_{S1}^{2}\right)}{\left(M_{S2}^{2}-M_{S1}^{2}\right)}. \end{array}$$

If *M*_11_ > 0 and *M*_22_ < 0, *Ω* would be greater than zero and *f*_even_ > *f*_0_ > *f*_odd_. The inherent TZ is at the upper stopband. In contrast, if *M*_11_ < 0 and *M*_22_ > 0, *Ω* would be greater than zero and *f*_even_ < *f*_0_ < *f*_odd_. The inherent TZ is at the lower stopband. Thus, the inherent TZ is always on the same side of the passband with the even-mode resonant frequency [15], as shown in Figure 2.

With regard to the proposed TSSLRs, the location of the inherent TZ relates to the even-mode, which can be changed by the value of *L*_8_. The TZ shifts from the lower stopband to the upper stopband when *L*_8_ increases from 5.6 mm to 7.1 mm, as shown in Figure 2. In this study, the inherent TZ is located at the upper stopband due to *f*_even_ > *f*_0_ > *f*_odd_, which leads to a very good selectivity.

**Figure 5:**
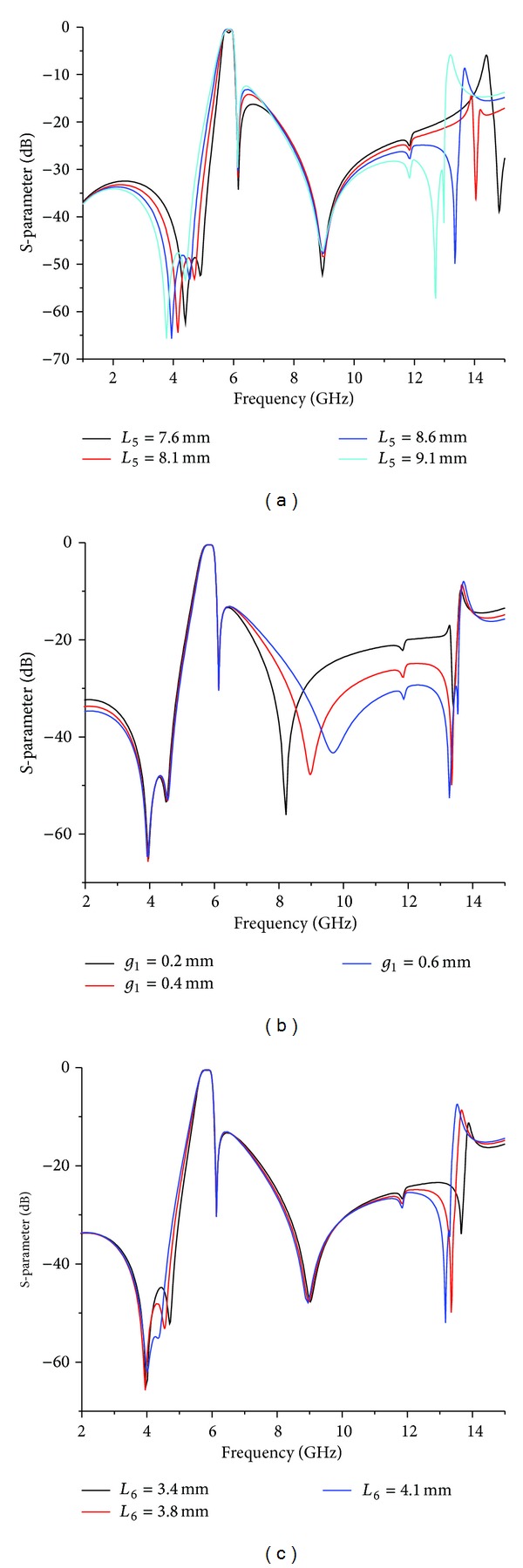
Simulated responses of the proposed BPF for different values of (a) *L*_5_, (b) *g*_1_, and (c) *L*_6_.

Except for the inherent TZ, four additional TZs are created by introducing two S-L couplings at both the lower and upper stopbands, which results in a wide stopband and a high second harmonic suppression. At the same time, the location of these four TZs can be adjusted by the amount of capacitance or inductance of the S-L coupling, as demonstrated in Figure 5.

As shown in Figure 5, the gap *g*_1_ determines the capacitance of the S-L coupling, whereas the coupling lengths *L*_5_ and *L*_6_ determine the inductance of the S-L coupling.  Figure 5(b) shows the simulated results of the proposed BPF with varied values of *g*_1_. When the value of *g*_1_ decreases, the additional TZ_4_ moves to the passband. Similarly, Figures 5(a) and 5(c) show the simulated responses of the proposed BPF with varied values of *L*_5_ and *L*_6_, respectively. When the values of *L*_5_ and *L*_6_ change, the TZ_2_ and TZ_3_ are shifted very little, while the other TZs are moved. Consequently, the inherent TZ and the four additional TZs can be controlled to achieve a good selectivity and high level of rejection at the stopband due to the modification of the amount of nonadjacent coupling capacitance and inductance.

## 3. Results and Discussion

**Figure 6:**
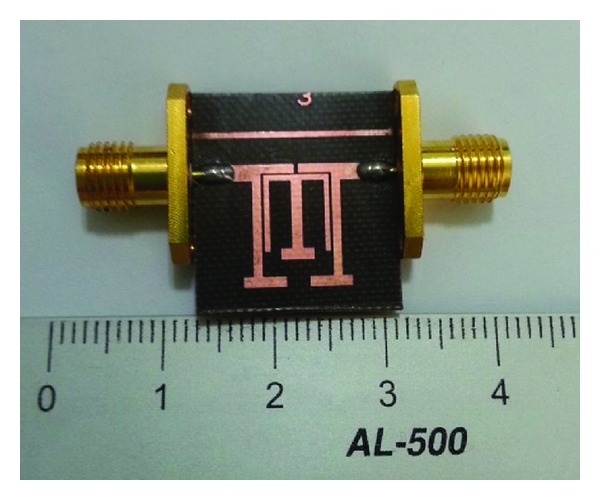
Photograph of the fabricated dual-band BPF.

Based on the proposed TSSLRs, a dual-mode BPF is fabricated using a Teflon substrate with a relative dielectric constant of 2.54, a thickness of 0.54 mm, and a loss tangent of 0.002. Following the preceding design process, the dimensions are determined as follows: *L*_1_ = 3.75 mm, *L*_2_ = 1.95 mm, *L*_3_ = 3 mm, *L*_4_ = 6.4 mm, *L*_5_ = 8.6 mm, *L*_6_ = 3.8 mm, *W*_1_ = 1.5 mm, *W*_2_ = 1 mm, *W*_3_ = 1 mm, *W*_4_ = 0.9 mm, *g*_1_ = 0.4 mm, and *g*_2_ = 0.2 mm, with an overall size of 7.9 × 11.1 mm^2^, as shown in Figure 6.

**Figure 7:**
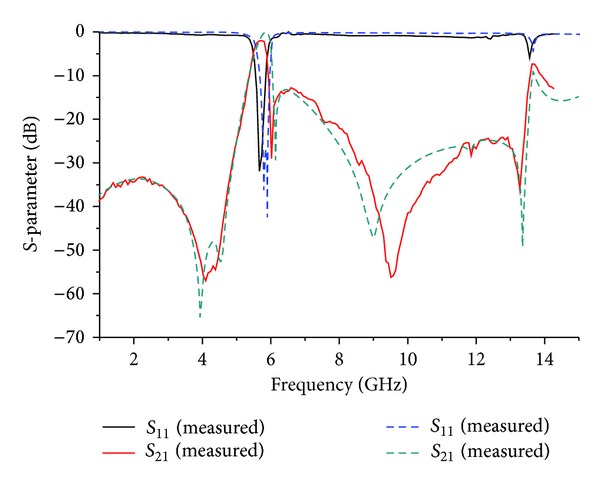
Simulation and measurement results of the proposed BPF with the proposed TSSLR.

The fabricated BPF was tested and characterized using an Agilent 8510C vector network analyzer (VNA). The narrow-band simulation and measurement results of the proposed BPF are demonstrated in Figure 7. As seen from the measurement results, the resonant frequency is exactly at 5.73 GHz, with an insertion loss of 1.75 dB, a return loss of 28.18 dB, and a FBW of 8.4%. Five controllable TZs are located at 4.08 GHz, 4.38 GHz, 6.02 GHz, 9.5 GHz, and 13.27 GHz, with attenuation level of more than 30 dB. The last two attenuation poles can be shifted near the second harmonic frequency, which gives this BPF a wide stopband and high rejection level.

## 4. Conclusion

In this study, a miniaturized narrow-band BPF using proposed TSSLRs is designed, simulated, and fabricated. The performance of the TSSLRs was studied using the odd-even-mode method and verified by a full-wave EM simulator. The proposed TSSLRs have an inherent TZ, and two modified feed-lines are used to generate more TZs at the stopband for high level rejection and harmonic suppression. The measured responses show that this BPF exhibits five TZs due to the introduced capacitive and inductive S-L couplings. The proposed BPF has potential to be applied in C-band RFID and WiMax applications.
